# Varicose Veins and Risk of Venous Thromboembolic Diseases: A Two-Sample-Based Mendelian Randomization Study

**DOI:** 10.3389/fcvm.2022.849027

**Published:** 2022-04-14

**Authors:** Ruihao Li, Zuoguan Chen, Liang Gui, Zhiyuan Wu, Yuqing Miao, Qing Gao, Yongpeng Diao, Yongjun Li

**Affiliations:** ^1^Department of Vascular Surgery, Beijing Hospital, National Center of Gerontology, Institute of Geriatric Medicine, Chinese Academy of Medical Sciences, Beijing, China; ^2^Graduate School of Peking Union Medical College, Beijing, China

**Keywords:** Mendelian randomization, varicose veins, deep vein thrombosis, pulmonary embolism, venous thromboembolism

## Abstract

**Background:**

Varicose veins are found to be associated with increased risk of venous thromboembolism (VTE) in many observational studies, but whether varicose veins are causally associated with VTE remains unclear. Therefore, we used a series of Mendelian randomization (MR) methods to investigate that association.

**Methods:**

23 independent single-nucleotide polymorphisms (SNPs) for varicose veins were obtained from the Pan UK Biobank analysis. The outcomes datasets for deep vein thrombosis (DVT), pulmonary embolism (PE) and venous thromboembolism (VTE) were obtained from the FinnGen study. Before analysis, body mass index (BMI) and height were included as confounders in our MR model. Basic MR [inverse-variance weighted (IVW), weight-median, penalized weighted-median and MR-Egger methods] and MR-PRESSO were performed against each outcome using the whole SNPs and SNPs after excluding those associated with confounders. If causal associations were suggested for any outcome, a basic MR validation analysis, a multivariable MR analysis with BMI and height, a Causal Analysis Using Summary Effect estimates (CAUSE), and a two-step MR analysis with BMI and height, would follow.

**Results:**

Using 21 qualified SNPs, the IVW method (OR: 1.173, 95% CI: 1.070–1.286, *p* < 0.001, FDR = 0.002), the weighted median method (OR: 1.255, 95% CI: 1.106–1.423, *p* < 0.001, FDR = 0.001), the penalized weighted median method (OR: 1.299, 95% CI: 1.128–1.495, *p* < 0.001, FDR = 0.001) and the MR-PRESSO (OR: 1.165, 95% CI: 1.067–1.273, *p* = 0.003, FDR = 0.009) suggested potential causal effect of varicose veins on DVT, but no cause effect was found for PE and VTE. Excluding SNPs associated with confounders yielded similar results. The causal association with DVT was validated using a self-reported DVT cohort (IVW, OR: 1.107, 95% CI: 1.041–1.178, *p* = 0.001). The causal association maintained after adjustment for height (OR = 1.105, 95% CI: 1.028–1.188, *p* = 0.007), BMI (OR = 1.148, 95% CI: 1.059–1.244, *p* < 0.001) and them both (OR = 1.104, 95% CI: 1.035–1.177, *p* = 0.003). The causal association also survived the strict CAUSE (*p* = 0.018). Finally, in two-step MR, height and BMI were found to have causal effects on both varicose veins and DVT.

**Conclusion:**

Genetically predicted varicose veins may have a causal effect on DVT and may be one of the mediators of obesity and taller height that predispose to DVT.

## Introduction

Varicose veins are an important manifestation of chronic venous disease (CVD), and they can be present thought nearly the whole course of CVD from small varicosities to venous ulcer (1). Varicose veins are generally recognized as a weak risk factor of venous thromboembolic diseases, i.e., deep vein thrombosis (DVT), pulmonary embolism (PE) and venous thrombosis (VTE) (2, 3). For patients receiving surgical procedures, the presence of lower extremity varicose veins adds one point to the total Caprini score that may result in VTE prophylaxis upgrade.

Several observational studies have examined the association between varicose veins and the risk of VTE. A cross-sectional study reported 8-fold increased odds of DVT in 2,357 patients documented with varicose veins in German population (4). A recent population-based longitudinal study revealed 4-fold increased risk of DVT in patients with varicose veins during a median follow-up of 7.5 years (5). Besides, varicose veins have also been found to confer additional DVT risk to patients who already had high VTE risk factors, e.g., cancer and orthopedic surgery (6, 7). However, to our best knowledge, no study has tested whether the risk of VTE can be reduced if varicose veins are surgically removed or ablated. Therefore, it is of considerable clinical interest to investigate whether varicose veins are causally associated with VTE (3).

Previous epidemiological studies on varicose veins and VTE were susceptible to confounding factors, making causal inference difficult. By contrast, Mendelian randomization (MR) uses genetic variations as instrumental variables to mimic a randomized trial, and is capable of uncovering the causal relationship between exposures and outcomes (8, 9). MR has been wildly carried out to test causalities in the field of cardiovascular research (10). For example, MR analysis revealed that genetically determined alcohol consumption, at all dose, increases the risk of coronary heart disease and hypertension (11, 12). In the present study, we utilized a series of two-sample-based MR methods to explore whether there are causal associations between varicose veins and venous thromboembolic diseases.

## Methods

### Exposure and Outcome Datasets

Genetic variants, i.e., single-nucleotide polymorphisms (SNP), were chosen as instrumental variables (IV). Genome-wide association study (GWAS) summary statistics for varicose veins of European ancestry were obtained from the Pan-ancestry Genetic Analysis of the UK Biobank adjusted for age, sex, age^*^sex, age2, age2^*^sex and the first 10 principal components (https://pan.ukbb.broadinstitute.org/). A total of 1,567 SNPs reached the genome-wide significance threshold (*P* < 5E-8), of which 23 appeared to be independent after clumping (based on the 1,000 genomes reference panel for Europeans, r2 = 0.001, kb = 10,000) for linkage disequilibrium. The SNPs were searched in the PhenoScanner database (http://www.phenoscanner.medschl.cam.ac.uk/) for associated genes and phenotypes other than varicose veins. The details of the 23 SNPs were listed in Table 1 and Supplementary Table 1. The strength of each of the IVs were evaluated by R^2^ statistic (the proportion of variance explained) and F statistic. R^2^ was calculated with formula 2 × MAF × (1–MAF) × (β estimate in SD units)^2^, whereas F statistic was calculated from R^2^ as F = (N – K – 1)/K × R^2^/(1 – R^2^) (13). MAF refers to minor allele frequency, K is the number of selected IVs and N is the samples size. A F statistic > 10 indicates a strong IV.

**Table 1:** Details of SNPs selected as instrumental variables.

**SNP**	**Effect allele**	**Other allele**	**Beta**	**SE**	***P*** **-value**	**EAF**	**Genes**
rs11121615	T	C	−0.284	0.015	6.55E-81	0.690	CASZ1
rs72787716	T	C	−0.098	0.017	4.72E-09	0.209	LBH
rs6546368	C	T	0.155	0.014	3.59E-27	0.657	AC017083.3
rs2734045	A	G	0.100	0.014	2.21E-13	0.482	LINC01565
rs28558138	C	G	−0.131	0.014	2.52E-21	0.420	snoU13
rs2250127	A	G	0.144	0.016	5.74E-20	0.247	LINC01184
rs11135046	T	G	−0.121	0.014	4.97E-19	0.543	EBF1
rs1155207	G	A	−0.106	0.014	7.46E-15	0.487	HIST1H3PS1
rs62401797	G	C	−0.146	0.026	1.09E-08	0.079	RP11-228O6.2
rs2800709	G	T	−0.078	0.014	7.91E-09	0.519	RSPO3
rs75731123	G	A	−0.108	0.019	1.28E-08	0.149	RP1L1
rs10817784	G	A	0.094	0.016	1.49E-09	0.738	DEC1
rs2083714	G	A	0.081	0.014	1.92E-09	0.499	SBF2
rs55726902	A	G	0.100	0.016	2.08E-10	0.242	HDAC7
rs41286076	T	C	0.092	0.015	3.03E-09	0.256	KLF5
rs4772697	G	A	0.083	0.014	3.59E-09	0.361	RP11-252M24.1
rs437564	T	C	0.085	0.014	2.60E-09	0.377	RP11-1348G14.4
rs34457921	G	A	−0.088	0.015	4.88E-09	0.298	ZFPM1
rs2911463	A	G	−0.190	0.015	1.07E-37	0.687	PIEZO1
rs236548	A	G	−0.119	0.016	3.16E-14	0.745	CALM2P1
rs2241173	G	A	−0.091	0.014	3.15E-11	0.575	LINC01152
rs6021277	T	C	0.102	0.014	8.16E-14	0.460	NFATC2
rs6062619	G	A	−0.113	0.016	3.84E-13	0.268	SOX18

*EAF, effective allele frequency; SE, standard error; SNP, single-nucleotide polymorphism*.

The outcome summary statistics for venous thromboembolic diseases were obtained from the 5th release of the FinnGen study (https://r5.finngen.fi/, Supplementary Table 2). The respective datasets for lower extremity DVT, PE, and VTE were “I9_PHLETHROMBDVTLOW” (4,576 cases and 190,028 controls), “I9_PULMEMB” (4,185 cases and 214,228 controls), and “I9_VTE” (9,176 cases and 209,616 controls). The cases were identified through either hospital discharge or cause of death ICD codes attached to the disease. Since all analyses were based on publicly available summary statistics derived from biobanks which had already been approved by their local ethical committees, no further ethical approval was required.

### MR Model and Study Design

There are three key assumptions for genetic variants to be valid. First, the genetic variants should be associated with exposure. Second, the genetic variant should not be associated with confounders of the exposure-outcome relationship. Third, the genetic variants exert effects on the outcome only via the exposure (8). However, the second and third assumption are often violated since most genetic variants are actually pleiotropic. Therefore, beyond conventional MR analyses, we conducted a series of robust MR analyses, including Mendelian randomization pleiotropy residual sum and outlier (MR-PRESSO) (14), multivariable MR (15), Causal Analysis Using Summary Effect estimates (CAUSE) (16) to account for pleiotropic effects.

**Figure 1:**
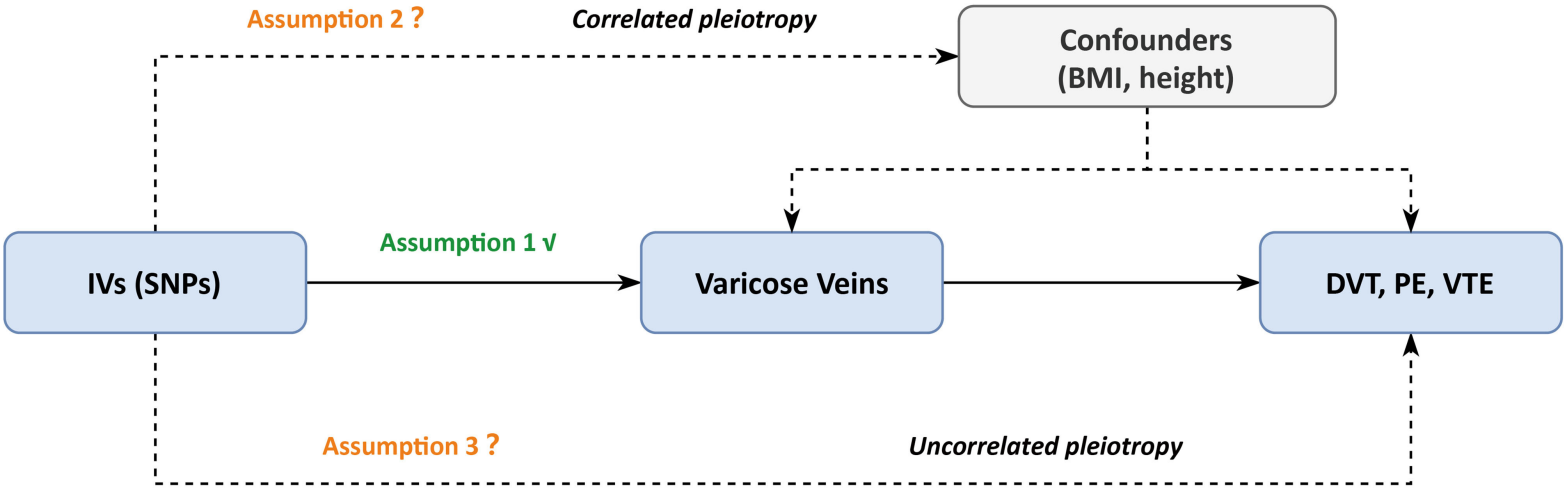
Mendelian randomization model in this study. BMI, body mass index; DVT, deep vein thrombosis; IV, instrumental variables; PE, pulmonary embolism; SNP, single-nucleotide polymorphism; VTE, venous thromboembolism. Assumption 1: the genetic variants should be associated with exposure. Assumption 2: the genetic variant should not be associated with confounders of the exposure-outcome relationship. Assumption 3: the genetic variants exert effects on the outcome only via the exposure.

**Figure 2:**
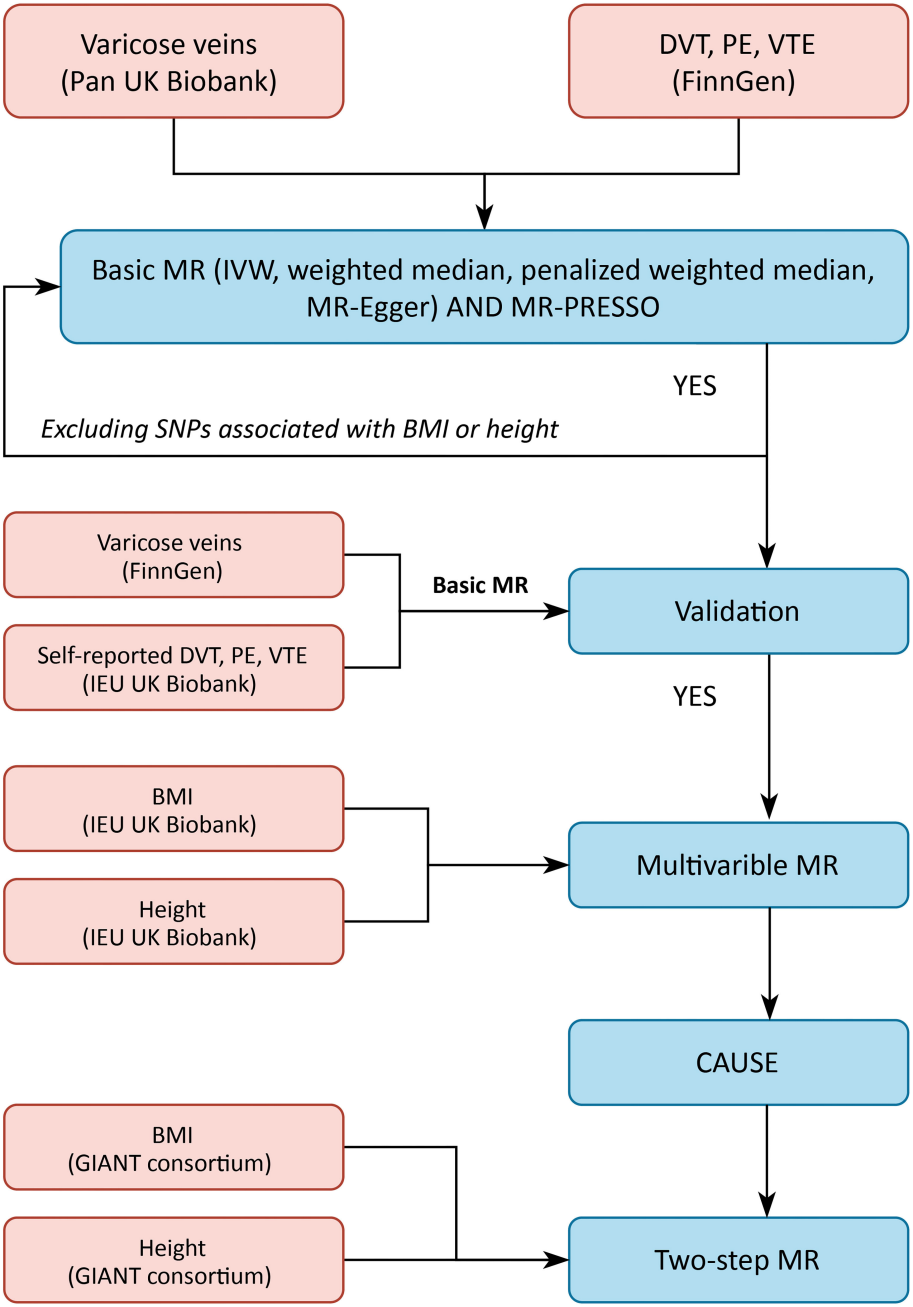
Flow of the study. CAUSE, Causal Analysis Using Summary Effect estimates; IVW, inverse-variance weighted; MR, Mendelian randomization; MR-PRESSO, Mendelian randomization pleiotropy residual sum and outlier.

As shown in the Supplementary Table 1, these SNPs also displayed genome-wide associations with other traits that mainly enriched in anthropometric and blood cell parameters, causing potential IV assumption violations. Taller height (17–19) and elevated BMI (20–23) are well-established risk factors for venous thromboembolic diseases according to both clinical observational and genetic association studies. In addition, obesity is a tradition risk factor (24) and taller height is a newly discovered risk factor (25, 26) of varicose veins. Given these clinical and genetic clues, we included these two traits as potential confounders in the varicose veins and VTE relationship in our MR model, and the following MR methods were applied to test whether there was a true causality between varicose veins and VTE. The illustrations of our MR model and its MR solutions were shown in Figures 1, 2. It is worth noting that only causality suggested by basic MR analysis would undergo further tests.

### Basic MR and MR-PRESSO

For basic MR analysis, we chose the inverse-variance-weighted (IVW) method as the main method, and the weighted-median (WM) method, the penalized-weighted-median (PWM) methods and the MR-Egger regression method as supplementary analyses (27). The IVW method gives unbiased estimates if all the instrumental variables are valid or directional pleiotropic is absent (28). The WM method provides a consistent estimate if at least half of the instrumental variables are valid, but may be less efficient (27). The PWM method down-weights the outlying variants most while affects the other variants at minimum (27). The MR-Egger method allows one or more genetic variants to have pleiotropic effects, as long as the size of these pleiotropic effects is independent of the size of the genetic variants' effects on the exposure (29). In addition, we applied MR-PRESSO test to identify horizontal pleiotropic outliers (14). After excluding SNPs concomitantly associated with height or BMI, we re-performed basic MR analysis and MR-PRESSO.

### MR Using Validation Cohorts

The FinnGen study also contains GWAS summary statistics of varicose veins (https://r5.finngen.fi/, I9_VARICVE), which may serve as another source of IVs. Besides, self-reported venous outcomes are available in the UK Biobank cohort, too (https://gwas.mrcieu.ac.uk/, ukb-b-12040). By interchanging the two cohort, basic MR analysis as mentioned above was used to validate any causal associations suggested by the primary datasets (details available in Supplementary Table 2).

### Multivariable MR

Multivariable MR is an extension to conventional MR that uses genetic variants associated with multiple, potentially related exposures to estimate the effect of each exposure on a single outcome (15). This approach gives unbiased causal estimates of direct effect if confounders are adjusted for (15, 30). Summary statistics for height and BMI were obtained from the IEU Open GWAS Project (https://gwas.mrcieu.ac.uk/, Supplementary Table 2). We conducted three rounds of multivariable MR analyses: varicose veins against the outcomes adjusted for (1) height alone, (2) BMI along, and (3) height and BMI combined. Because the primary exposure is not included in the IEU Open GWAS Project and automatic data formatting is not possible, we manually formatted the data for multivariable MR analyses. In brief, independent SNPs for each exposure were collected and combined, and then the essential information (i.e., betas, standard errors, *p*-values and effect allele frequencies) for the combined SNPs were extracted from each exposure's GWAS summary statistics.

### MR With CAUSE

There are two kinds of horizontal pleiotropy, one is uncorrelated pleiotropy and the other is correlated pleiotropy. Uncorrelated pleiotropy occurs when a genetic variant affects the outcome and the exposure of interest through separate mechanisms (violation of the third IV assumption), whereas correlated pleiotropy occurs when a genetic variant affects the outcome and the tested exposure through a shared heritable factor (violation of the second IV assumption) (16). In this case, correlated pleiotropy may occur because of height and BMI, leading to false positive. Therefore, CAUSE developed by He et al. (16) were utilized to account for both correlated and uncorrelated pleiotropic effects. In brief, complete GWAS summary statistics of the exposure and the outcomes were merged, and nuisance parameters were calculated using one million unique variants for the merged data. Finally, nuisance parameters and top variants after LD pruning were used to fit CASUSE. We performed CAUSE following the online instruction with default parameters for LD pruning.

### Two-Step MR

Once varicose veins were proved to be causally associated with one or more venous thromboembolic diseases, it was of interest to investigate whether varicose veins play as mediators between height and/or BMI and VTE. Therefore, two-step MR was utilized to assess potential mediation effects (31). Genetic instrumental variables for height and BMI were obtained from the Genetic Investigation of Anthropometric Traits (GIANT) Consortium via the IEU Open GWAS Project (https://gwas.mrcieu.ac.uk/, Supplementary Table 2). In the first step, the genetic variants of height and BMI were used to perform MR analysis (IVW method) against varicose veins. And in the second step the genetic variants were used to perform MR analysis against venous thromboembolic diseases. Mediation effects were suggested if evidence of causalities appeared in both steps.

### Statistical Analysis

All analyses were conducted using R version 4.1.1 (The R Foundation for Statistical Computing, Vienna, Austria) under Windows environment. The R packages for MR analyses were “TwoSampleMR” (https://mrcieu.github.io/TwoSampleMR/index.html), “MR-PRESSO” (https://github.com/rondolab/MR-PRESSO) and “CAUSE” (https://jean997.github.io/cause/). A *p* two-sided *p*-value lower than 0.05 indicated statistical significance and supported a causal relationship. In addition, false discovery rate (FDR) adjusted *p*-values proposed by Benjamini and Hochberg were used to address multiple hypotheses testing (32).

## Results

### Strength of Selected Genetic Variables

The total variance explained by these SNPs were 15.0% (Supplementary Table 3), which is similar to a variance-explained of 13.4% based on 12 SNPs in a previous GWAS (26) of the same cohort. The mean and total F statistics were 120.92 and 2,871.55, respectively, indicating strong IVs.

### Basic MR and MR-PRESSO

Among 23 selected SNPs derived from the exposure dataset, 19 were initially matched with the outcome datasets. 3 LD proxied SNPs were qualified (r2 > 0.9) for the missing SNPs and were used for harmonization (Supplementary Table 4). rs34457921 were excluded from harmonization for no qualified proxy and rs28558138 was exclude for being palindromic, leaving 21 SNPs for basic MR analysis and MR-PRESSO.

**Figure 3:**
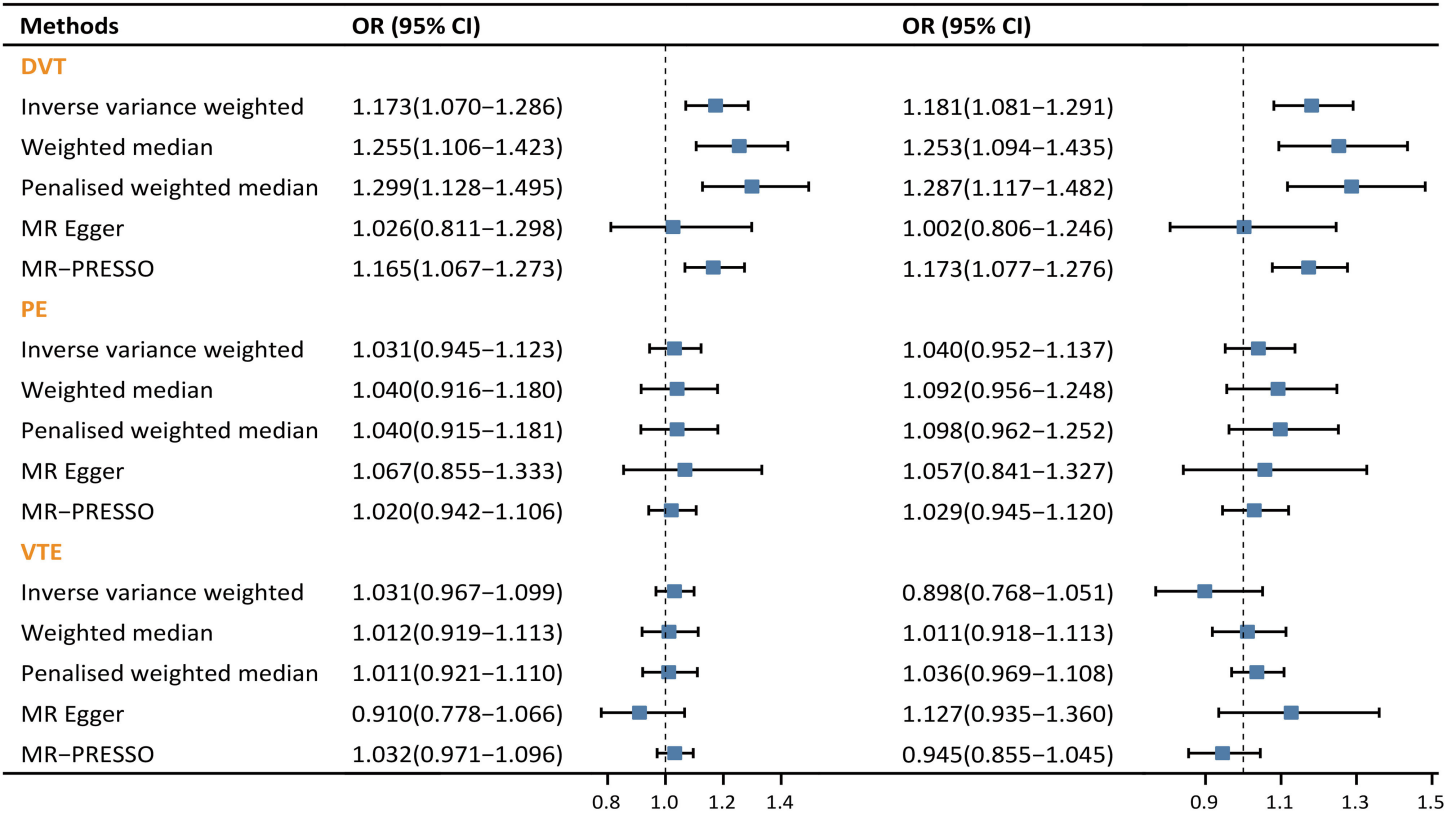
Forest plots of causal effect of varicose vein on venous thromboembolic diseases. **(Left)** MR analysis using all 21 qualified SNPs. **(Right)** MR analysis using 19 SNPs that have no genome-wide association with confounders. CI, confidence interval; OR, odds ratio.

For DVT, a causal association was suggested using the IVW method (OR: 1.173, 95% CI: 1.070–1.286, *p* < 0.001, FDR = 0.002), the weighted median method (OR: 1.255, 95% CI: 1.106–1.423, *p* < 0.001, FDR = 0.001) and the penalized weighted median method (OR: 1.299, 95% CI: 1.128–1.495, *p* < 0.001, FDR = 0.001), the MR-PRESSO (OR: 1.165, 95% CI: 1.067–1.273, *p* = 0.003, FDR = 0.009), with the exception of the MR-Egger method (OR: 1.026, 95% CI: 0.811–1.298, *p* = 0.830). The effect sizes and their corresponding CIs of basic MR analyses were illustrated in Figure 3 (left). No significant directional pleiotropic effect was detected by the Egger-intercept test (intercept = 0.018, *p* = 0.241). Besides, no pleiotropic outlier was detected by MR-PRESSO. Details of other supporting statistics were listed in Supplementary Table 5. Since rs41286076 and rs1155207 were associated with height, we excluded them and re-performed basic MR analysis, and similar results were obtained (Figure 3, right). The scatter plots, forest plots, and leave-one-out plots for DVT were available in Supplementary Figure 1.

However, as for PE and VTE, no sign of causal association was observed in either basic MR analysis or MR-PRESSO. Non-significant effect estimates remained even after excluding those SNPs associated with height (Figure 3). As a result, PE and VTE were excluded from further MR analyses.

### Validation

Using the validation cohort where the DVT outcome was self-reported, the IVW (OR: 1.107, 95% CI: 1.041–1.178, *p* = 0.001), the WM method (OR: 1.126, 95% CI: 1.033–1.226, *p* = 0.007) and the PWM method (OR: 1.127, 95% CI: 1.036–1.227, *p* = 0.005) consistently showed that varicose veins were causally associated with DVT, whereas the MR-Egger method suggested no association (OR: 1.157, 95% CI: 0.956–1.400, *p* = 0.144). Scatter plot is available in Supplementary Figure 2.

### Multivariable MR

**Figure 4:**
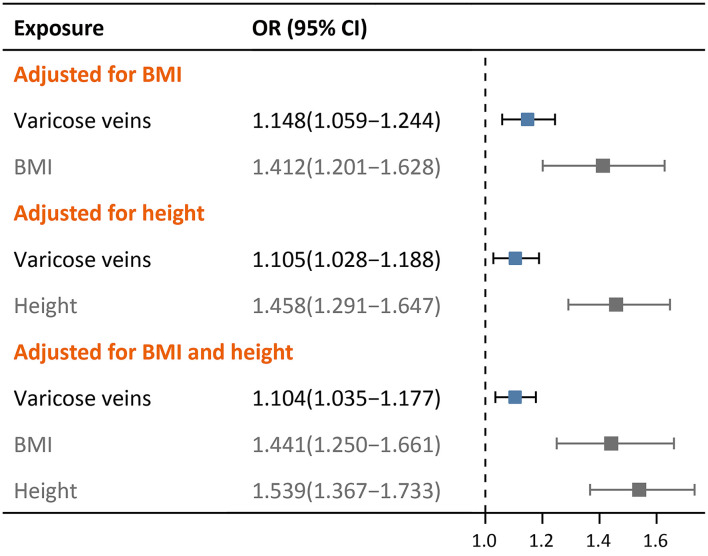
Forest plots of multivariable MR adjusted for BMI and/or height.

In multivariable MR (Figure 4), varicose veins consistently showed a causality with DVT after adjustment for height (OR = 1.105, 95% CI: 1.028–1.188, *p* = 0.007), BMI (OR = 1.148, 95% CI: 1.059–1.244, *p* < 0.001) and both of them (OR = 1.104, 95% CI: 1.035–1.177, *p* = 0.003). The effect sizes of causal association slightly attenuated in multivariable MR as compared with univariable MR.

### CAUSE

In the strict CAUSE, the causal model was shown to be a better fit than the sharing model (*p* = 0.018), indicating a causal association between varicose veins and DVT. More supporting statistics were listed in Supplementary Table 6.

### Two-Step MR

**Figure 5:**
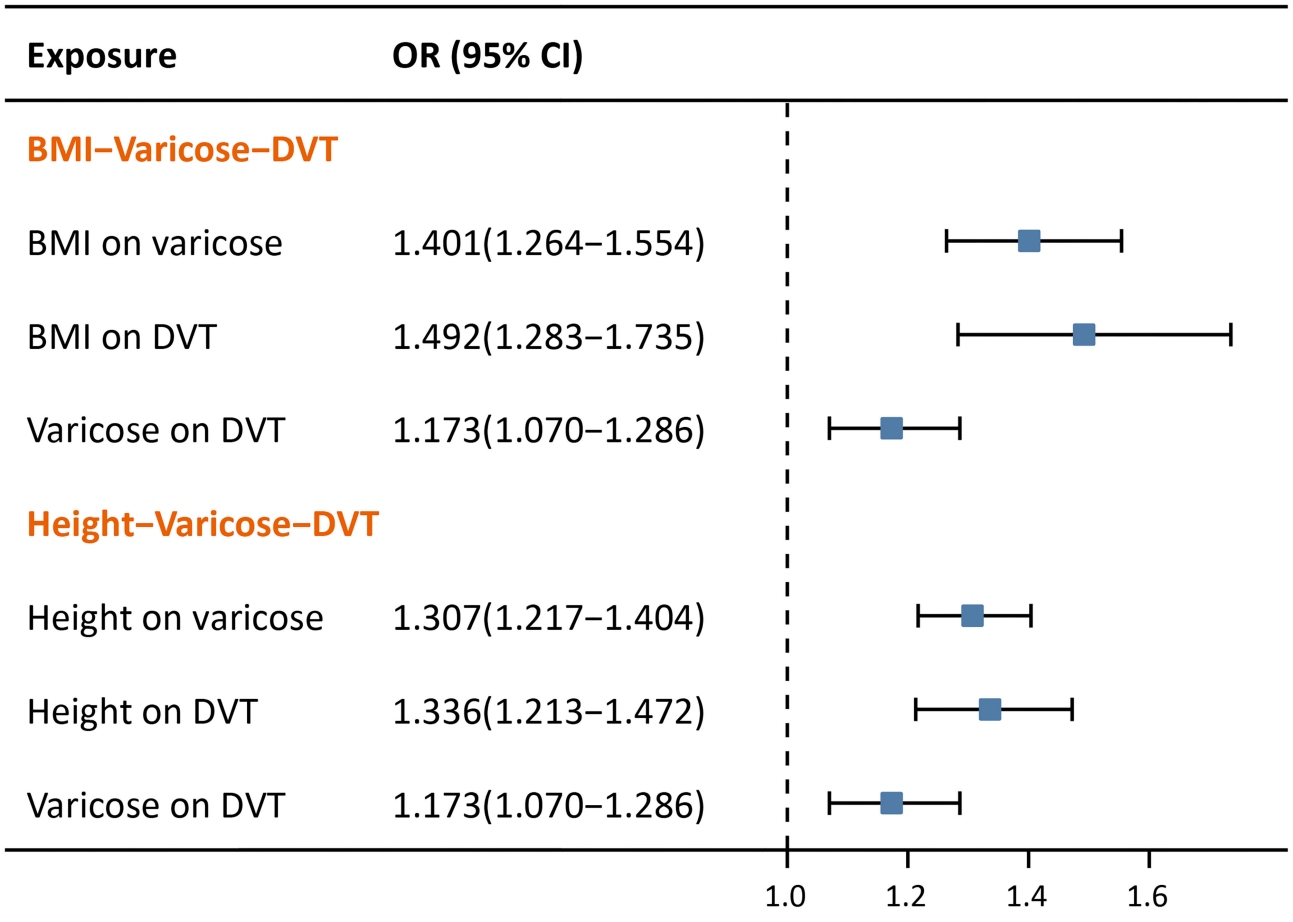
Forest plots of two-step MR with BMI and height.

As expected, in two-step MR (Figure 5), BMI was shown to be casually associated with both varicose veins (OR = 1.401, 95% CI: 1.264–1.554, *p* < 0.001) and lower extremity DVT (OR = 1.492, 95% CI: 1.283–1.735, *p* = *p* < 0.001). Similarly, taller height was also found to have casual associations with both varicose veins (OR = 1.307, 95% CI: 1.217–1.404, *p* < 0.001), and lower extremity DVT (OR = 1.336, 95% CI: 1.213–1.472, *p* < 0.001).

## Discussion

In this comprehensive MR study, our results highlighted that genetically predicted varicose veins may have a causal association with DVT and may be one of the mediators of traditional DVT risk factors that predispose to DVT, e.g., obesity and taller height. Our findings concur with previous observational studies that varicosity is an independent risk factor of DVT (4–6, 33), but are less susceptible to unmeasured confounders.

The causal associations were, however, non-significant for PE and VTE. Since DVT is seen as the most common cause of PE, a causal risk factor of DVT is also considered a causal risk factor for PE. Several reasons may explain this paradox in our studies. At first, the effect size and of varicose veins on PE would be smaller than that of DVT because PE is the downstream of DVT, and the CI would be wider as well. Furthermore, the percentage of DVT and PE overlap was low in the FinnGen study (9.1%), which means that the causal effect of varicose veins on PE via DVT may have been too small to be detected using MR methods. In fact, the difference between DVT and PE is more remarkable than previous thought (34). For example, patients carrying the factor V Leiden mutation are reported to have a substantially increased risk to develop DVT but only a mildly increased risk to acquire PE (35). In contrast, several risk factors like pneumonia, COPD, atrial fibrillation, and sickle cell disease lead to a higher risk of PE and seem to have a much smaller effect on DVT (36, 37). Similar to the FinnGen study, many studies have reported high rates of isolated PE in the absence of DVT (20–79.3%), and patients with isolated PE are more likely to have cancer, atrial fibrillation and heart failure and be exposed to hormone therapy (37–41). One recent meta-analysis concluded that PE is not associated with lower extremity DVT in adult trauma patients (42). Furthermore, one study used comprehensive magnetic resonance imaging to detect origins of pulmonary emboli, but could only find a origin in less than half of the patients, suggesting PE may arise *de novo* in the lungs (*in-situ* thrombosis) (40). These evidences indicate that DVT and PE have differences in risk factors, etiology and pathophysiology and these differences may have genetic backgrounds. Hence, it was possible that no causal association were found for the remaining two outcomes. Future releases of biobank-level GWAS with larger sample sizes are need to clarify these issues.

In an early population-based case-control study, varicose veins were found to be an independent risk factor of VTE in an age-dependent manner, with people aged 45 suffering the highest risk (OR: 4.19) (33). In another observation confined to DVT, the presence of varicose veins was associated with 8 times the odds of DVT in German population (4). In one case-control study enrolling patients aged over 70 years, the adjusted odds ratio for VTE patients to have varicose veins was 1.6 (95% CI: 1.2–2.3) (43). And more recently, Chang et al. (5) found the hazard ratios for developing DVT and PE in varicose vein cases were 5.3 and 1.73, respectively, as compares with non-varicose vein cases in a 7.5-year long follow-up. Despite the findings form epidemiological investigations, studies aiming to elucidate the mechanisms underlying the risk difference of DVT were scarce. One possible explanation could be that the turbulent flow and venous stasis cause by reflux in primary venous insufficiency predisposes to a prothrombotic state of lower extremity (44). Another hypothesis is about chronic inflammation. Some inflammatory and prothrombotic markers (e.g., IL-6, TNF-α, vWF, and PAI-1) has been reported to be significantly elevated in varicose veins (45). And that it is well-recognized that inflammation is an important trigger of thrombosis (46). However, no direct biological evidence has been found that varicose veins are causes of DVT, therefore, the exact role of varicose veins in the occurrence of DVT is under-explored. An external mechanism easy to think of is that varicose vein surgeries lead to the increased risk of DVT, but the Chang et al.'s investigation found that the magnitudes only slightly attenuated after excluding those who received varicose vein surgeries (47), indicating potential mechanisms from within.

In contrast, the mechanism by which a DVT lead to varicose veins is clearer. Venous obstruction and valvular reflux may appear after a chronic DVT event, causing persistent venous hypertension and finally leading to post-thrombotic syndrome (PTS), of which varicose veins is an important manifestation (48). Our bi-directional MR analysis also supported that DVT has a causal effect on varicose veins (IVW, OR: 1.111, 95% CI: 1.040–1.187, *p* = 0.002), which corroborated previous study (25).

The strength of our studies is obvious, that the MR methods were robust and comprehensive, and the MR model was designed from a clinical perspective. However, several limitations of the studies should be mentioned as well. First, although primary varicose veins and secondary varicose veins (e.g., PTS) differ in genetics and pathophysiology, they might be coded under the same diagnostic code in real world settings (44). Due to a lack of individual-level data, we cannot exclude PTS cases in the exposure dataset, and thus the results may not completely represent the VTE risks associated with primary varicose veins. Nonetheless, one smaller GWAS of varicose veins (26) using the UK Biobank cohort had adjusted for DVT and BMI and yielded 13 qualified IVs, still the MR result supported a causal association between varicose veins and DVT (IVW, OR: 1.184, 95% CI: 1.083–1.293). Second, the outcomes were also based on ICD codes without further verification, and coding errors may lead to misclassification of diseases as previous observational studies (4, 5). To remedy this, we chose self-reported DVT cohort as supplement, and the primary finding was reproductive using the basic MR methods. Third, varicose veins affect much more women than men, but current datasets were not sufficient to conduct a gender-specific MR analysis. Fourth, all the study population were of European ancestry in our study but the disease patterns may vary across different ancestries, therefore, generalizing the finding to non-European ancestries may not be suitable. Given these limitations, the causal association between varicose vein and DVT suggested by our MR analyses should be interpreted with caution. More studies are warranted to better clarify the causal association between varicose veins and VTE.

## Conclusion

In conclusion, using a series of robust MR methods, we found that genetically determined varicose veins may have causal effect on DVT. In addition, we revealed that varicose veins may serve as mediators of obesity and taller height on the increased risk of DVT. Our study brings some new insight into the relationship between varicose veins and DVT, and future basic experiments or well-designed clinical studies are warranted to corroborate these findings.

## Data Availability Statement

The datasets presented in this study can be found in online repositories. The names of the repository/repositories and accession number(s) can be found in the article/Supplementary Material.

## Ethics Statement

The studies involving human participants were reviewed and approved by Local Ethics Committees of the FinnGen Project and the UK Biobank Project, no additional ethical approval are required. The patients/participants provided their written informed consent to participate in these study.

## Author Contributions

RL: conception, design, data analysis and interpreting, and writing. ZC and LG: conception, data analysis, and revision. ZW and YM: data analysis and interpreting. QG: data interpreting. YD: critical revision. YL: conception and critical revision. Final approval was obtained from all authors.

## Funding

This work was support by a grant from Wu Jieping Medical Foundation (320.6750.19089-36).

## Conflict of Interest

The authors declare that the research was conducted in the absence of any commercial or financial relationships that could be construed as a potential conflict of interest.

## Publisher's Note

All claims expressed in this article are solely those of the authors and do not necessarily represent those of their affiliated organizations, or those of the publisher, the editors and the reviewers. Any product that may be evaluated in this article, or claim that may be made by its manufacturer, is not guaranteed or endorsed by the publisher.
