# Supplementary material for: Varicose Veins and Risk of Venous Thromboembolic Diseases: A Two-Sample-Based Mendelian Randomization Study
Source: Front Cardiovasc Med. 2022 Apr 14;9:849027. doi: 10.3389/fcvm.2022.849027 (PMC9047357; doi:10.3389/fcvm.2022.849027)
Supplement: Supplementary file 1 [file Data_Sheet_1.docx]

Supplementary Material

# Supplementary Table

**Supplementary Table 1.** Details of varicose vein instrumental variables and possible associations with other traits

| **SNP** | **Other traits** |
| --- | --- |
| rs11121615 | Impedance of leg, leg fat-free mass, whole body fat-free mass, leg predicted mass, whole body water mass |
| rs72787716 | NA |
| rs6546368 | NA |
| rs2734045 | Monocyte percentage of white cells, monocyte count, basophil count, impedance of leg left, sum eosinophil basophil counts |
| rs28558138 | Self-reported hypertension, diastolic blood pressure |
| rs2250127 | Red cell distribution width, impedance of leg, leg predicted mass, impedance of whole body, leg fat percentage, whole body fat-free mass, whole body water mass, basal metabolic rate, trunk fat-free mass, cellulitis, lymphocyte count, forced vital capacity, body fat percentage, immature fraction of reticulocytes |
| rs11135046 | Breast cancer, systolic blood pressure |
| rs1155207 | Mean corpuscular hemoglobin, mean corpuscular volume, height, red cell distribution width, hemoglobin concentration, disorders of mineral metabolism, trunk fat mass, hematocrit, weight, whole body fat mass, basal metabolic rate, hip circumference adjusted for BMI, leg predicted mass, body fat percentage, whole body water mass, whole body fat-free mass, waist circumference, arm fat-free mass, diastolic blood pressure, forced vital capacity, primary sclerosing cholangitis |
| rs62401797 | NA |
| rs2800709 | Heel bone mineral density, hemoglobin concentration, red blood cell count, hematocrit, hip circumference, reticulocyte count |
| rs75731123 | Vascular or heart problems diagnosed by doctor |
| rs10817784 | NA |
| rs2083714 | Diastolic blood pressure, systolic blood pressure, trunk predicted mass, trunk fat-free mass, red blood cell count, impedance of whole body, impedance of left, hemoglobin concentration, hematocrit |
| rs55726902 | Allergic disease asthma hay fever or eczema, platelet count, plateletcrit, allergic disease, self-reported hypertension |
| rs41286076 | Height |
| rs4772697 | NA |
| rs437564 | Mean corpuscular volume, red blood cell count |
| rs34457921 | NA |
| rs2911463 | Mean corpuscular hemoglobin concentration, hemoglobin concentration, reticulocyte count, red blood cell count, reticulocyte fraction of red cells |
| rs236548 | Sitting height |
| rs2241173 | NA |
| rs6021277 | NA |
| rs6062619 | NA |

Abbreviations: BMI, body mass index; NA, not available; SNP, single-nucleotide polymorphism.

**Supplementary Table 2.** Details of the all datasets included in the studies.

| **Phenotype** | **Source** | **Dataset** | **Unit** | **No. cases** | **No. controls** | **Definition** | **Purpose** |
| --- | --- | --- | --- | --- | --- | --- | --- |
| Varicose veins | Pan-UK Biobank | I83 European | log OR | 12059 | 408472 | ICD-10: I83 | Main exposure dataset |
| Lower extremity DVT | FinnGen | I9_PHLETHROMBDVTLOW | log OR | 4576 | 190028 | ICD-10: I80.20, I80.29, I80.3  ICD-9: 4511, 4510, 4512  ICD-8: 4510 | Main outcome dataset |
| PE | FinnGen | I9_PULMEMB | log OR | 4185 | 214228 | ICD-10: I26  ICD-9: 415  ICD-8: 450 | Main outcome dataset |
| VTE | FinnGen | I9_VTE | log OR | 9176 | 209616 | ICD-10: I26, I80, O87.1, O88.2  ICD-9: 415, 451, 6713, 6714, 6732  ICD-8: 450, 451, 671, 6739 | Main outcome dataset |
| Varicose veins | FinnGen | I9_VARICVE | log OR | 17027 | 190028 | ICD-10: I83  ICD-9: 454  ICD-8: 454 | Exposure dataset for validation |
| DVT | MRC-IEU analysis of UK Biobank | ukb-b-12040 | Transformed log OR | 9241 | 453692 | Self-reported | Outcome dataset for validation |
| **Phenotype** | **Source** | **Dataset** | **No. variants** | **No. participants** | | **Definition** | **Purpose** |
| Standing height | MRC-IEU analysis of UK Biobank Consortium | ukb-b-10787 | SD | 461460 | | - | Multivariable MR |
| BMI | MRC-IEU analysis of UK Biobank | ukb-b-19953 | SD | 461460 | | - | Multivariable MR |
| Height | GAINT consortium | ieu-a-89 | SD | 253288 | | - | Two-step MR |
| BMI | GAINT consortium | ieu-b-40 | SD | 681275 | | - | Two-step MR |

**Supplementary Table 3.** Strength of selected genetic instrumental variables.

| **SNP** | **Beta** | **SE** | **EAF** | **R^2^** | **F statistic** |
| --- | --- | --- | --- | --- | --- |
| rs11121615 | -0.284 | 0.015 | 0.690 | 0.035 | 653.390 |
| rs72787716 | -0.098 | 0.017 | 0.209 | 0.003 | 58.241 |
| rs6546368 | 0.155 | 0.014 | 0.657 | 0.011 | 200.136 |
| rs2734045 | 0.100 | 0.014 | 0.482 | 0.005 | 91.754 |
| rs28558138 | -0.131 | 0.014 | 0.420 | 0.008 | 154.149 |
| rs2250127 | 0.144 | 0.016 | 0.247 | 0.008 | 142.120 |
| rs11135046 | -0.121 | 0.014 | 0.543 | 0.007 | 133.822 |
| rs1155207 | -0.106 | 0.014 | 0.487 | 0.006 | 103.223 |
| rs62401797 | -0.146 | 0.026 | 0.079 | 0.003 | 56.887 |
| rs2800709 | -0.078 | 0.014 | 0.519 | 0.003 | 55.705 |
| rs75731123 | -0.108 | 0.019 | 0.149 | 0.003 | 54.241 |
| rs10817784 | 0.094 | 0.016 | 0.738 | 0.003 | 62.687 |
| rs2083714 | 0.081 | 0.014 | 0.499 | 0.003 | 60.174 |
| rs55726902 | 0.100 | 0.016 | 0.242 | 0.004 | 67.322 |
| rs41286076 | 0.092 | 0.015 | 0.256 | 0.003 | 59.138 |
| rs4772697 | 0.083 | 0.014 | 0.361 | 0.003 | 58.294 |
| rs437564 | 0.085 | 0.014 | 0.377 | 0.003 | 62.261 |
| rs34457921 | -0.088 | 0.015 | 0.298 | 0.003 | 59.430 |
| rs2911463 | -0.190 | 0.015 | 0.687 | 0.016 | 288.323 |
| rs236548 | -0.119 | 0.016 | 0.745 | 0.005 | 98.903 |
| rs2241173 | -0.091 | 0.014 | 0.575 | 0.004 | 74.298 |
| rs6021277 | 0.102 | 0.014 | 0.460 | 0.005 | 94.990 |
| rs6062619 | -0.113 | 0.016 | 0.268 | 0.005 | 92.057 |
| Total |  |  |  | 0.150 | 2781.546 |

**Supplementary Table 4.** Missing SNPs and proxies.

| **SNP** | **Proxy** | **Chr** | **Position** | **Distance (kb)** | **r^2^** | **Qualified?** |
| --- | --- | --- | --- | --- | --- | --- |
| rs2734045 | rs2713587 | 3 | 128290848 | 1212 | 1 | Yes |
| rs6546368 | rs7579440 | 2 | 68498508 | 324 | 0.987 | Yes |
| rs10817784 | rs7033287 | 9 | 118287525 | 2582 | 0.975 | Yes |
| rs34457921 | rs28703652 | 16 | 88620640 | 6354 | 0.886 | No |

Abbreviations: Chr, chromosome; SNP, single-nucleotide polymorphism.

URL: <https://analysistools.cancer.gov/LDlink/?tab=ldproxy>

**Supplementary Table 5. Supporting statistics of basic MR and MR-PRESSO.**

| **Sensitive parameter** | **DVT** | **PE** | **VTE** |
| --- | --- | --- | --- |
| MR-Egger Q | 22.502 | 18.309 | 19.634 |
| MR-Egger p | 0.260 | 0.502 | 0.417 |
| IVW Q | 24.24 | 18.421 | 22.552 |
| IVW p | 0.232 | 0.560 | 0.311 |
| Egger-intercept | 0.018 | -0.005 | 0.017 |
| Egger-intercept p | 0.241 | 0.741 | 0.109 |
| MR-PRESSO outlier | None | None | None |

**Abbreviations:** DVT, deep vein thrombosis; PE, pulmonary embolism; VTE, venous thromboembolism.

**Supplementary Table 6. Supporting statistics of CAUSE.**

| **Comparison** | **Model 1** | **Model 2** | **Delta_elpd** | **Se_delta_elpd** | **Z** |
| --- | --- | --- | --- | --- | --- |
| 1 | Null | Sharing | -5.311 | 2.360 | -2.250 |
| 2 | Null | Causal | -9.401 | 4.217 | -2.229 |
| 3 | Sharing | Causal | -4.090 | 1.945 | -2.103 |

# Supplementary Figures


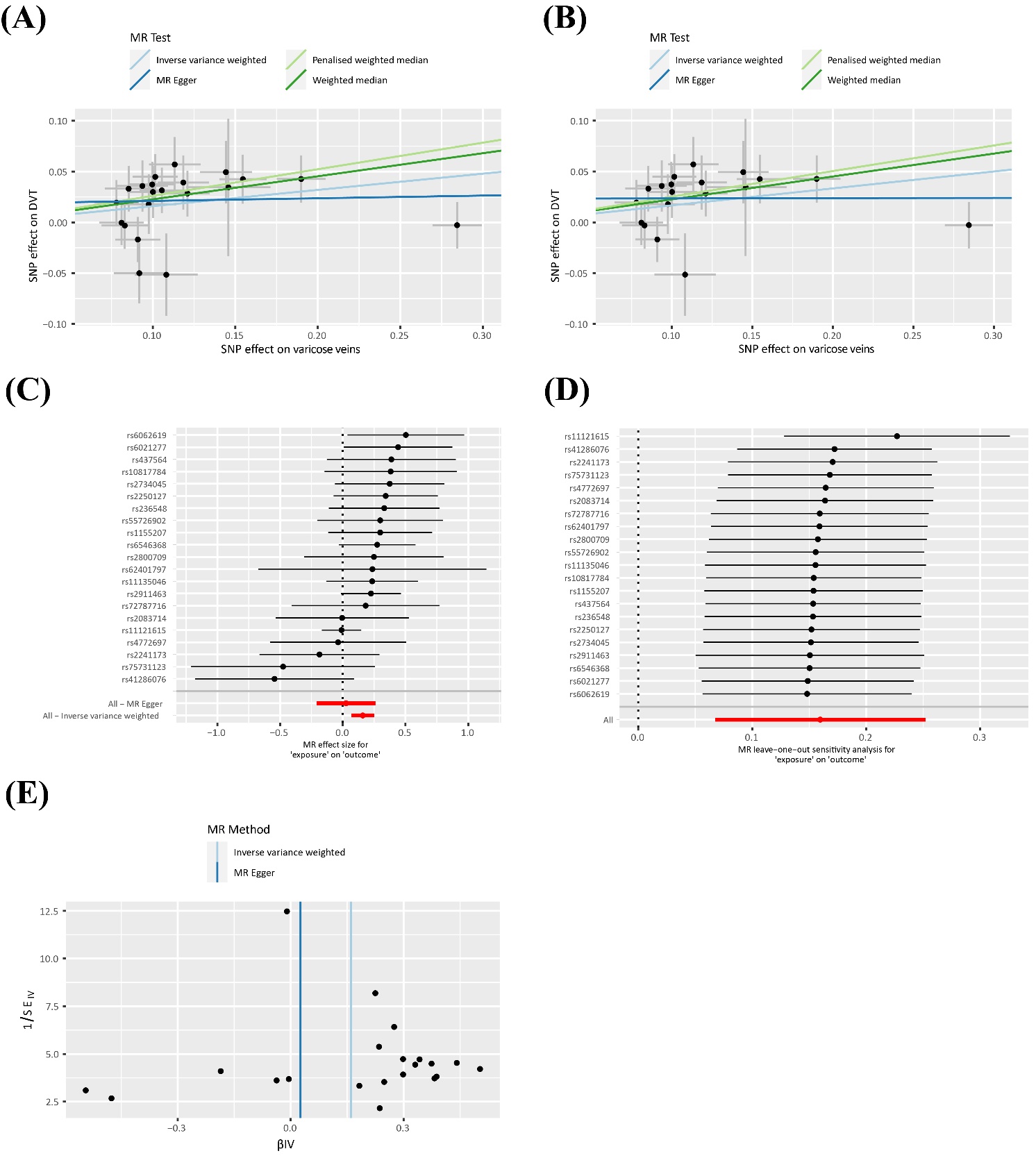


**Supplementary Figure 1.** Scatter plots, forest plot, leave-one-out plot and funnel plot of causal effect of varicose veins on DVT. (A) Scatter plot using all 21 SNPs. (B) Scatter plot using 19 SNPs that have no association with confounders. (C) Forest plot of effect SNPs on DVT. (D) Leave-one-out plot. (E) Funnel plot for IVW method and MR-Egger.


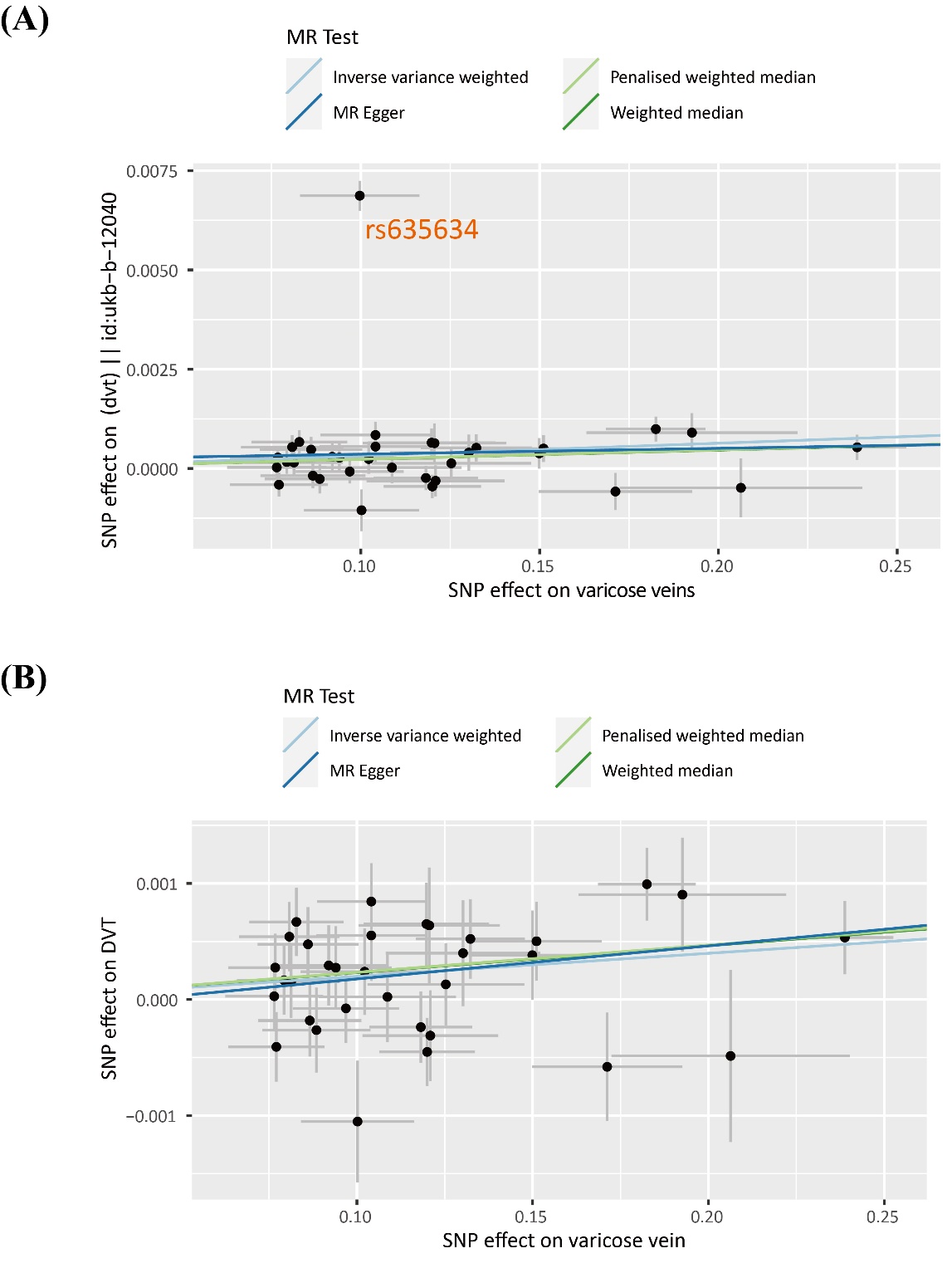


**Supplementary Figure 2.** Scatter plot of causal effect of varicose veins on DVT using validation cohorts. (A) When the outlier rs635634 was not excluded. (B) When the outlier was excluded.
